# Early combined rehabilitation intervention to improve the short-term prognosis of premature infants

**DOI:** 10.1186/s12887-021-02727-8

**Published:** 2021-06-09

**Authors:** Yang Liu, Zheng-feng Li, Yun-huan Zhong, Zhi-hui Zhao, Wen-xin Deng, Ling-ling Chen, Bei-bei Liu, Tao-jun Du, Yong Zhang

**Affiliations:** 1 Department of Neonatology, Sichuan Provincial Maternity and Child Health Care Hospital, No. 290 West Second Street, Shayan Road, Chengdu, 610031 Sichuan China; 2 Sichuan Provincial Maternity and Child Health Care Hospital, Chengdu, 610031 Sichuan Province China

**Keywords:** Early rehabilitation comprehensive intervention treatment, Premature infants, Prognosis

## Abstract

**Background:**

To explore the clinical effect of early combined rehabilitation intervention on premature infants in the neonatal intensive care unit (NICU).

**Methods:**

Premature infants with gestational ages less than 32 weeks or birth weights less than 1500 g were included in the present study.The participants were divided into the intervention group and control group. All infants received the current routine treatment based on the clinical guidelines, and the intervention group was additionally treated by visual and auditory stimulation, oral motor function, respiratory function and neurodevelopmental training. The following clinical outcomes were compared: durations of oxygen supplementation and indwelling gastric tube use; incidences of retinopathy of prematurity (ROP) and neonatal necrotizing enterocolitis (NEC); Sliverman scores; incidences of bronchopulmonary dysplasia (BPD) and intraventricular haemorrhage; days of hospitalization; and neurodevelopmental outcomes. Datas were analysed using the following statistical tests: the chi-square test, the independent samples or paired t test, repeated measures ANOVA, and the Wilcoxon rank sum test.

**Results:**

Compared with those in the control group, premature infants in the intervention group had shorter durations of oxygen supplementation and indwelling gastric tube use, fewer hospitalization days and lower incidences of ROP, BPD, and NEC.The intervention group had lower Sliverman scores and higher Ballard neuromuscular scores than the control group.

**Conclusion:**

Early combined rehabilitation intervention can improve the short-term clinical outcomes of premature infants.

## Background

With the development of assisted reproductive technology and perinatal medicine, the birth rate and survival rate of preterm infants have gradually increased. However, the short-term and long-term morbidities of premature infants present a noticeable disease burden. Premature infants, especially very premature and very-low-birth-weight babies, have a high incidence of neurological disability. Previous studies have shown that cerebral palsy occurs in 5 to 10% of premature infants, motor disturbances occur in 25 to 40% of premature infants, and cognitive, attentional, behavioural, and socialization disturbances occur in 25 to 50% of premature infants, with a likelihood of problems with visual perception and visual-motor function as well as visual and hearing impairments [1–6].

Rehabilitation treatment is an important way to improve prognosis [7]. A previous study proposed that combined rehabilitation treatment, including the Bobath method, ultrasonic therapy devices and transcutaneous electrical stimulators, nutritional nerve drugs and family intervention, was effective for premature infants with brain injury at 40 weeks postmenstrual age (PMA) [8].

Hadiseh Ghomi proposed that early oral motor intervention is an effective method of rehabilitation [9]. Manuela Filippa suggests that early sound contact and music are beneficial for the brain development of premature infants [10]. Katherine Ross’s findings demonstrated that neonatal therapy can be initiated early in gestation, and the rehabilitation interventions mentioned in this study include neuromotor and oral motor function [11]. We advocate early rehabilitation intervention, but the specific programme has not yet been unified.

Therefore, here, we introduce the concept of early combined rehabilitation interventions, including visual, auditory, oral motor, respiratory function and neuromotor function interventions, to explore the clinical effect of early combined rehabilitation interventions on premature infants in the NICU.

## Methods

### Study design and participants

The study was conducted from April 2019 to October 2019 in the NICU of the neonatology department of the affiliated hospital of Sichuan Provincial Maternity and Child Health Care Hospital.

The inclusion criteria were as follows: gestational age less than 32 weeks, weight less than 1500 g, and consent by the parents for the infant to undergo early comprehensive rehabilitation intervention.

The exclusion criteria were congenital malformations, genetic metabolic diseases, severe asphyxia, and lack of consent by the parents for the infant to undergo the intervention.

A total of 51 premature infants were included in the study according to the inclusion and exclusion criteria. The study was divided into groups according to whether their parents agreed to carry out early comprehensive rehabilitation intervention.Finally, there were 22 cases in the treatment group and 29 cases in the observation group. This study was reviewed and approved by the hospital’s medical ethics committee of Sichuan Provincial Maternity and Child Health Care Hospital. The verbal informed consent was obtained from the parents of all children, the ethics committee approved this procedure.

### Intervention

We began the comprehensive rehabilitation intervention when the infants had stable respiration and oxyhemoglobin saturation under non-invasive ventilation, and the intervention ended at discharge. This rehabilitation treatment was evaluated and implemented by professionally trained physical therapists in our hospital. The frequency is once a day, and the total time is approximately 20–25 min. All infants received the current routine treatment based on the clinical guidelines, and the intervention group was additionally treated by visual and auditory stimulation, oral motor function, respiratory function and neurodevelopment training. Vital signs were monitored during the whole treatment process, and we would stop immediately if the signs were not stabilized.

#### Visual and auditory intervention

Red balls were used to gently shake the infants’ eyes before limb treatment to attract attention and stimulate the child’s tracking ability. In the whole therapy, music was played to stimulate the children’s tracking and hearing ability for approximately 20–25 min.

**Respiratory intervention** (divided into three steps)

1. The thinking behind using Rood therapy involves using a brush stimulation technique, with cotton swabs, to stimulate the patient’s chest breathing according to the muscle group and to stimulate a spontaneous breathing response for a duration of 5 min.
2. Chest compression and vibration techniques are used to prevent the collapse of the trachea and bronchus and maintain tension of the tube wall, and these measures are applied for approximately 5 min [12].
3. With tactile stimulation, the physical therapist touches and massages the child’s limbs, face, chest and abdomen by hand and stimulates the normal development of the baby’s original reflex and muscle tone for approximately 5 min.

#### Neuromotor intervention

Bobath and Vojta are used to train the infants with passive and active exercises according to their postures, muscular tension, and reflexes for approximately 10 min to promote the development of neuromotor function in the child.

#### Oral motor intervention

A cotton swab is moistened with normal saline, and the cotton swab is used to wipe the skin from the outer cheek to the root of the ear, the area between the nose and upper lip, the inner cheek side of the mouth and the upper and lower gums and the lips, forming a “circle” shape. A cotton swab is used to stimulate the tongue and the lower part of the tongue, promote the tip of the tongue to stick out of the mouth, lift the tongue several times, press lightly with the hand, and stimulate the front of the ear and the cheek for 2–3 min.

### Evaluation of the efficacy of the early combined rehabilitation intervention

**Fig. 1:**
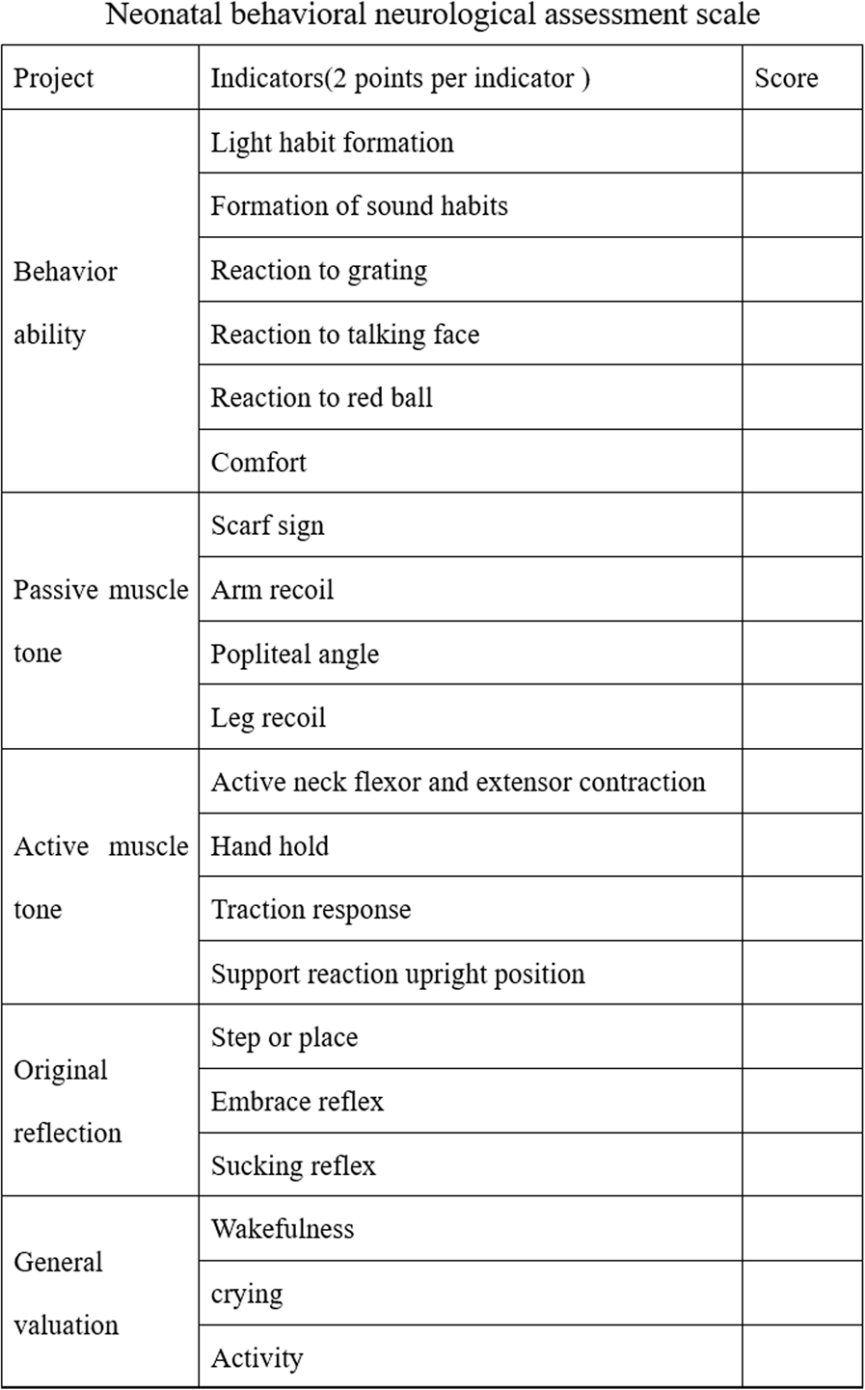
Neonatal behavioral neurological assessment scale.

1. Clinical data: the data collected included the duration of oxygen supplementation and indwelling gastric tube use, common complications of preterm infants (ROP, NEC, BPD, intracranial haemorrhage), and days of hospitalization.
2. Sliverman scoring scale: The score was evaluated according to five aspects: upper chest movement, lower chest movement, xiphoid depression, nose instigation, and groaning during exhalation. Scores of 0–3 were classified as no or slight breathlessness, and scores of 4–6 were classified as moderate breathlessness. Scores of 7–10 indicated difficulty breathing and were classified as severe dyspnoea; specifically, the lower the Sliverman score was, the less dyspnoea there would be [13].
3. Neuromuscular/neurological signs in the Ballard score: Sings in the Ballard score include behavioural ability (response to a red ball and sound), muscle tone (active and passive muscle tone: square window sign, upper limb rebound, popliteal angle, scarf sign, and ear sign), primitive reflex (hand grip, foot grip, asymmetrical tonic neck reflex (ATNR)), and posture. The higher the neuromuscular score was, the better the development would be [13].
4. neonatal behavioral neurological assessment(NBNA)Scale: Premature infants were evaluated for neurobehavioural scoring using the 20-item neurobehavioural neuroscore method when their gestational age was PMA 40 weeks. The measurement was performed in a quiet, semidark room at a temperature of 24–26 °C 1 h after breastfeeding, and the total inspection time was 10 min. Interval inspections were performed when the status of the infant was not good. Diagnostic criteria: neonatal NBNA basic item score < 35 points indicated brain injury, and 35–36 points indicated suspected brain injury [14].(Fig. 1)

### Statistical analysis

The data were analysed using descriptive statistics and the following statistical tests: the chi-square test, the independent samples or paired t test, repeated measures ANOVA, and the Wilcoxon rank sum test. All analyses were performed using SPSS, version 23. *P* < 0.05 indicated a significant difference.

## Results

There was no statistically significant difference in sex, gestational age, birth weight, or start time of the intervention between the intervention group and the control group (Table 1).

**Table 1:** Demographic data comparison (Mean ± SD^a^)

Group	Cases	Sex (Cases)		Birth weight (g)	Gestational age (weeks)	Intervention time (PMA, weeks)
		Man	Male			
The intervention group	22	11	11	1348.18 ± 207.10	30.3 ± 1.1	32 ± 1.2
The control group	29	13	16	1338.45 ± 211.51	30.7 ± 1.3	31 ± 1.5
*F/χ2-*value		0.134		0.027	0.908	2.613
*P*-value		0.714^b^		0.87 ^b^	0.345 ^b^	0.112 ^b^

^a^Standard deviation

^b^ There was no statistically significant difference in sex, gestational age, birth weight, or start time of the intervention between the intervention group and the control group

### Early combined rehabilitation intervention reduces the durations of oxygen supplementation and indwelling gastric tube use and the days of hospitalization

The time of invasive ventilation before starting intervention was not statistically significant between the two groups.The durations of oxygen supplementation in the intervention group and control group were 22.84 ± 13.78 days and 31.98 ± 15.97 days, respectively, and the difference was statistically significant (*F* = 4.596, *P* < 0.05). The durations of the indwelling gastric tube in the intervention group and control group were 23.13 ± 12.84 days and 33.45 ± 15.48 days, respectively, and the difference was statistically significant (*F* = 6.596, *P* < 0.05). The days of hospitalization in the intervention group and control group were 39.18 ± 10.32 days and 47.45 ± 16.11 days, respectively, and the difference was statistically significant (*F* = 4.406, *P* < 0.05) (Table 2).

**Table 2:** Comparison of clinical treatment indicators (Mean ± SD, day)

Group	Invasive ventilation time	Noninvasive ventilation time	duration of oxygen supplementation	duration of indwelling gastric tube use	Days for hospitalization
Intervention group	3.63 ± 8.60	13.60 ± 9.08	22.84 ± 13.78	23.13 ± 12.84	39.18 ± 10.32
control group	5.55 ± 9.98	21.47 ± 12.98	31.98 ± 15.97	33.45 ± 15.48	47.45 ± 16.11
*F*-value	0.534	5.881	4.596	6.596	4.406
*P*-value	0.469^d^	0.019 ^a^	0.037^a^	0.015 ^b^	0.041^c^

^abc^ The difference was statistically significant in the Noninvasive ventilation time,the duration of oxygen supplementation, the duration of indwelling gastric tube use,days for hospitalization between the intervention group and the control group

^d^ The time of invasive ventilation before starting intervention was not statistically significant between the two groups

### Early combined rehabilitation intervention reduces the incidence of ROP, BPD, and NEC among premature infants

The incidences of ROP in the intervention group and control group were 9 and 34%, respectively. All cases were in the first stage of ROP, and the difference was statistically significant *(χ2* = 3.183, *P* < 0.05). The incidence of BPD was 23% in the intervention group and 55% in the control group, and the difference was statistically significant *(χ2* = 8.065, P < 0.05). The incidence of NEC was 4.5% in the intervention group and 27.6% in the control group; The difference was statistically significant *(χ2* = 4.062, *P* < 0.05). The incidence of intracranial haemorrhage was 4.5% in the intervention group and 17.2% in the control group, and the difference was not statistically significant (*χ2* = 0.912, *P* > 0.05) (Table 3).

**Table 3:** Comparison of the incidence of common complications in preterm infants (case (%))

Group	ROP	BPD	NEC	intracranial haemorrhage
The intervention group	2 (9)	5 (23)	1 (4.5)	1 (4.5)
The control group	10 (34)	16 (55)	8 (27.6)	5 (17.2)
*χ2*-value	3.183	8.065	4.062	0.912
*P*-value	0.048^a^	0.005^b^	0.025^c^	0.34^d^

^abc^ The difference was statistically significant in the incidences of ROP,BPD,NEC in the intervention group and control group

^d^ The difference was not statistically significant in the incidence of intracranial haemorrhage between the intervention group and the control group

### The NBNA score

The NBNA score at PMA 40 weeks in the intervention group was 38.14 ± 0.94, and that in the control group was 37.52 ± 1.43. There was no significant difference between the two groups (*P* > 0.05). Further statistical analysis and the Wilcoxon rank sum test showed that the difference was statistically significant (*P* < 0.05) (Table 4).

**Table 4:** Comparison of NBNA scores

Group	scores > 37	scores = 35–36	scores < 35	*T*-value	*P*-value
The intervention group	21	1	0	17.66	0.000^b^
The control group	23	5	1		

^a^ There was no significant difference in the mean of NBNA scores between the two groups (*P* > 0.05)

^b^ Further statistical analysis and the Wilcoxon rank sum test showed that the difference was statistically significant (*P* < 0.05)

### Early combined rehabilitation intervention improves the degree of dyspnoea in premature infants

The Sliverman scores of the two groups at different times were significantly different (*F* = 89.071, *P* < 0.05), and there was an interaction between the test time and the group (*F* = 2.609, *P* = 0.037 < 0.05). Comparison of Sliverman scores in the intervention group and the control group at the beginning of the intervention showed no significant difference (*F* = 2.887, *P* > 0.05). The paired t test was used to compare each time period within the group, and the comparison of each time period within the group showed that the difference was statistically significant (*P* < 0.05); that is, with the passage of time, the Sliverman score gradually decreased. The scores of the intervention group in the first week, the second week, and the third week were all lower than those of the control group (*P* < 0.05); that is, the dyspnoea of the intervention group was lower than that of the control group. (Table 5).

**Table 5:** Comparison of Sliverman scores

Group	start time	first week	second week	third week	F-value	P-value
The intervention group	4.32 ± 1.91	2.36 ± 1.36	2.09 ± 1.15	1.41 ± 0.73	89.071	0.000^d^
The control group	5.17 ± 1.67	4.41 ± 1.78	3.48 ± 1.70	2.66 ± 1.91		
*F*-value	2.887	20.109	10.888	8.359	2.609	0.037^c^
*P*-value	0.096^a^	0.000^b^	0.000^b^	0.006^b^		

^a^ Sliverman scores in the intervention group and the control group at the beginning of the intervention group showed no significant difference (*P* > 0.05)

^b^ The comparison of each time period within the group showed that the difference was statistically significant (*P* < 0.05)

^c^ There was an interaction between the test time and the group (*P* < 0.05)

^d^ The Sliverman scores of the two groups at different times were significantly different (*P* < 0.05)

### Early combined rehabilitation intervention promotes neuromuscular motor development in premature infants

The neuromuscular scores in the Ballard score analysis of the two groups at different times were significantly different (*F* = 30.57, *P* < 0.05), and there was an interaction between the intervention group and the control group (*F* = 2.817, *P* < 0.05). There were no statistically significant differences in Ballard neuromuscular scores at the beginning of the intervention (*F* = 2.056, *P* > 0.05); the paired t test was used to compare each time period within the group, and the difference was statistically significant (*P* < 0.05); that is, as time went by, the Ballard neuromuscular score gradually increased. However, the scores of the intervention group in the first week, second week, and third week were higher than those of the control group (*P* < 0.05); that is, the neuromuscular score of the intervention group indicated that the muscle development in the intervention group was higher than that in the control group (Table 6).

**Table 6:** Comparison of the neuromuscular scores in the Ballard scores analysis

Group	start time	first week	second week	third week	F-value	*P*-value
The intervention group	17.23 ± 3.18	18.73 ± 1.72	20.00 ± 1.72	21.41 ± 1.87	30.571	0.000^d^
The control group	16.10 ± 2.43	16.24 ± 2.31	16.48 ± 2.20	17.97 ± 2.24		
*F*-value	2.056	17.896	34.446	38.446	2.817	0.049^c^
*P*-value	0.158^a^	0.000^b^	0.000^b^	0.000^b^		

^a^ Ballard scores in the intervention group and the control group at the beginning of the intervention group showed no significant difference(*P* > 0.05)

^b^ The comparison of each time period within the group showed that the difference was statistically significant (*P* < 0.05)

^c^ There was an interaction between the test time and the group (*P* < 0.05)

^d^ The Ballard scores of the two groups at different times were significantly different (*P* < 0.05)

## Discussion

The first 6 months after birth is the peak period of brain growth and development, and the infant nervous system at this stage has high plasticity [15]. The principle of early intervention is the plasticity of the brain, which is specifically manifested as learning and compensation. The brain has a strong ability to adapt and reorganize in structure and function and is easily affected by the environment [16–18] .Therefore, the infant can actively recover as soon as possible during this period. Intervention treatment can improve the prognosis of preterm infants. We chose the stable status of the preterm infants as the starting point for rehabilitation treatment, and the start date for the early combined rehabilitation intervention was gestational week PMA 32 ± 1.2 weeks.

In this study, rehabilitation interventions included a red ball chase, music therapy, touching, oral function training, respiratory function training, and neuromotor therapy. Research shows that NICU music exposure is beneficial to the cardiopulmonary system and is also beneficial to the hypothalamic-pituitary-adrenal axis, brain structure, and cognitive behavioural status [19, 20]. The combination of touch and increased visual stimulation may affect the maturity of cerebral cortex work, causing the infants in the intervention group to show more mature performance in spontaneous and targeted eye movements, tracking arcs, streak discrimination and distance attention [21]. Touch therapy and oral function training can increase body growth and promote the development of gastrointestinal function and neuromotor function for premature birth, promote coordination of neuromotor function and increase the frequency of bowel movements, thereby improving the oral feeding ability of preterm infants and improving prognosis [9, 22, 23]. Regarding neurodevelopmental results at 18–26 months, those who have a corrected the gestational age at 40 weeks and reach full-mouth feeding have a better neurological prognosis, with reduced cognition, language, motor delays, less cerebral palsy and fewer adverse neurodevelopmental results [24].As the duration of oxygen inhalation increases,release of proinflammatory cytokines has been implicated in the development of systemic inflammation that contributes to BPD and ROP.In this study, we provided Intervention program to reduce the duration of oxygen use, reduced the incidence of BPD and ROP, and conductted early assessment to predict long-term outcomes and guide later rehabilitation treatment [25, 26].

Early combined rehabilitation intervention can improve the short-term clinical outcomes of premature infants. Early combined rehabilitation intervention can promote the development of respiratory function and neuromuscular motor function, shorten the duration of oxygen supplementation and indwelling gastric tube use, reduce the incidences of ROP, NEC and BPD, and shorten the hospitalization time.

This study has certain limitations. It is a single-centre study with a small sample size and evaluated only relevant indicators during hospitalization. To further confirm our short-term results and the long-term prognosis of premature infants, we need to expand the sample size and closely follow up the discharged premature infants for further evaluation.

## Conclusion

Early combined rehabilitation intervention can improve the short-term clinical outcomes of premature infants.

## Data Availability

All the data and materials used are included in the manuscript. The datasets used and/or analysed during the current study available from the corresponding author on reasonable request.

## Abbreviations

NICU: neonatal intensive care unit
ROP: retinopathy of prematurity
NEC: neonatal necrotizing enterocolitis
BPD: bronchopulmonary dysplasia
ATNR: asymmetrical tonic neck reflex
PMA: postmenstrual age
NBNA: neonatal behavioral neurological assessment
